# Correspondence

**Published:** 1888-08

**Authors:** Robert Farley

**Affiliations:** Phœnixville, Pa.


### CORRESPONDENCE.

Editors Homœopathic Physician:—In answer to the queries of Dr. Hooker in July number of your journal, I reply as follows:

(1) "Is the disappearance of the immediate symptoms a sign of cure?" I did not claim it was, and do not wish to do so.

(2) "If so, why do the symptoms recur, in many instances, at the same time every year without a fresh exposure?" The answer to the first question will amply answer this one. But I may add, my statement was to the effect that nature was abundantly able in *some cases* to throw off the impression made by the poison in question, as I know from having been able to keep cases under observation for now nearly a score of years.

(3) "If Dr. F. was cured, why is he still so extremely susceptible to the poison?" I will answer this by asking Dr. H. a question: Why was I extremely susceptible from infancy, why were all my maternal ancestors also extremely susceptible, why are some of my brothers and sisters equally susceptible and others not at all, and why are some individuals so painfully susceptible to drug action in infinitesimal doses? The provings of Lac. can. give an excellent example of this susceptibility to something that "is only good to fatten puppies on."

(4) "Does the Doctor deny that the poison may be carried to the face and genitals by the unwashed hands as well as by systemic infection?" I did not so state. Dr. H. claimed actual contact was *the* cause of the increased involvement. I merely claimed that not to be so of a necessity, and probably very seldom the cause. If Dr. Hooker had read my statement carefully these queries would not have been called forth.

Robert Farley, M. D.

Phœnixville, Pa.

